# True and False Positive HIV Point of Care Test Results in a Prospective Multinational Study of At-Risk African Women: Implications for Large-Scale Repeat HIV Testing in HIV Prevention Programs

**DOI:** 10.1097/QAI.0000000000003497

**Published:** 2024-08-01

**Authors:** Susan Morrison, Joanne Batting, Valentine Wanga, Ivana Beesham, Jennifer Deese, G. Justus Hofmeyr, Margaret P. Kasaro, Cheryl Louw, Charles Morrison, Nelly R. Mugo, Thesla Palanee-Phillips, Melanie Pleaner, Krishnaveni Reddy, Caitlin W. Scoville, Jenni Smit, Jeffrey S.A. Stringer, Khatija Ahmed, Elizabeth Bukusi, Philip Kotze, Jared M. Baeten

**Affiliations:** a Department of Global Health, University of Washington, Seattle;; b Effective Care Research Unit, University of the Witwatersrand and Eastern Cape Department of Health, East London, South Africa;; c Foundation for Professional Development (FDP) Research Unit, East London, South Africa;; d Department of Epidemiology, University of Washington, Seattle;; e Department of Obstetrics and Gynaecology, MatCH Research Unit (MRU), Commercial City, Faculty of Health Sciences, University of the Witwatersrand, Durban, South Africa;; f Global Health, Population and Nutrition, FHI 360, Durham, NC;; g Currently, Global Respiratory Vaccines, Pfizer, Inc., Collegeville, PA;; h Eastern Cape Department of Health, Effective Care Research Unit, Universities of the Witwatersrand/Walter Sisulu;East London, South Africa;; i Department of Obstetrics and Gynecology, University of Botswana, Gaborone, Botswana;; j UNC Global Projects Zambia, Lusaka, Zambia;; k Department of Obstetrics and Gynecology, University of North Carolina Chapel Hill, School of Medicine, North Carolina;; l Madibeng Centre for Research, Brits, South Africa;; m Behavioral, Epidemiologic and Clinical Sciences, FHI 360, Durham, NC;; n Centre for Clinical Research, Kenya Medical Research Institute, Nairobi, Kenya;; o Wits Reproductive Health and HIV Institute, Faculty of Health Sciences, University of the Witwatersrand, Johannesburg, South Africa;; p MatCH Research Unit Edendale- Department of Obstetrics and Gynaecology, Faculty of Health Sciences, University of the Witwatersrand, Edendale, South Africa;; q Setshaba Research Centre, Soshanguve, South Africa;; r University of Pretoria, Department of Medical Microbiology, Pretoria, South Africa;; s Centre for Microbiology Research, Kenya Medical Research Institute, Nairobi, Kenya;; t Department of Obstetrics and Gynecology, University of Washington, Seattle;; u Qhakaza Mbokodo Research Clinic, Ladysmith, South Africa; and; v Department of Medicine, University of Washington, Seattle.

**Keywords:** HIV testing, HIV point of care test, false positive, true positive, African young women, prospective study

## Abstract

**Background:**

Accurate HIV point of care testing is the cornerstone of prevention and treatment efforts globally, although false (both negative and positive) results are expected to occur.

**Setting:**

We assessed the spectrum of true and false positive HIV results in a large prospective study of HIV incidence in African women using 3 contraceptive methods tested longitudinally in Eswatini, Kenya, South Africa, and Zambia.

**Methods:**

HIV serologic testing was conducted quarterly using 2 parallel rapid HIV tests. When one or both tests were positive, additional confirmatory testing was conducted, including HIV enzyme immunoassay (EIA) and RNA.

**Results:**

A total of 7730 women contributed 48,234 visits: true positive results occurred at 412 visits (0.9%) and false positives at 96 visits (0.2%). Of 412 women with HIV seroconversion, 10 had discordant (ie, 1 negative and 1 positive) rapid tests and 13 had undetectable HIV RNA levels. Of 62 women with false positive rapid HIV results, most had discordant rapid testing, but 6 (9.7%) had dually positive rapid results, and 4 (6.5%) had false positive or indeterminate EIA results. The positive predictive value of dual positive rapid results was 98.3%.

**Conclusions:**

Although most rapid test results were accurate, false positive results were expected and occurred in this population of initially HIV seronegative individuals tested repeatedly and prospectively. When HIV infection occurred, not all cases had textbook laboratory results. Our findings highlight the importance of confirmatory testing, particularly for individuals undergoing repeat testing and in settings where the point prevalence is expected to be low.

**Trial registration:**

ClinicalTrials.gov number NCT02550067.

## INTRODUCTION

HIV testing is the cornerstone of prevention and treatment efforts globally. For persons living with HIV, testing is the first step to linkage to care and antiretroviral therapy (ART) initiation. For persons who would benefit from HIV prevention services, including preexposure prophylaxis (PrEP), periodic testing is essential. In 2020, The Joint United Nations Programme on HIV/AIDS proposed targets for 2025 that 95% of persons living with HIV know their HIV status as part of a goal to end the global epidemic by 2030.^1^

Knowledge of HIV status is dependent on the availability and utilization of accurate HIV diagnostic testing. The diagnostic accuracy of a test is defined by its specificity and sensitivity. Historically, the emphasis has been placed on the ability of an HIV test to correctly identify all those who have the infection (sensitivity), as falsely negative tests potentially delay lifesaving ART. However, the ability of an HIV test to correctly identify all those without the condition (specificity) is less frequently discussed. An erroneous HIV diagnosis may lead to significant consequences for the individual^2^ such as avoidable experience of stigma and unnecessary initiation of lifelong ART.^3,4^ At a population level, cases of HIV misdiagnoses could undermine confidence in HIV testing.

The World Health Organization (WHO) recommends using an HIV testing strategy consisting of a combination of rapid diagnostic tests and/or enzyme immunoassays with ≥99% sensitivity and ≥98% specificity, which, when used together, achieve at least a 99% positive predictive value (PPV).^5^ However, PPV varies according to the prevalence of HIV in the community where the testing is performed. As the prevalence of HIV declines in a population, the fraction of positive test results that are true positives also falls. The advent of highly effective HIV prevention strategies such as PrEP potentially creates a novel scenario of mandatory periodic HIV testing among a population with low HIV incidence and thus decreased likelihood of a truly positive result.

We investigated the rates of true and false positive rapid HIV test results among women followed in a prospective study conducted in 4 countries in eastern and southern Africa, in which HIV acquisition was the primary end point. We conducted confirmatory, and if needed, additional advanced testing for all instances of positive HIV rapid test results, allowing clear definition of true and false positive testing.

## METHODS

### Population and Procedures

Participants were women aged 16–35 years enrolled in the Evidence for Contraceptive Options and HIV Outcomes trial at 12 research sites in Eswatini, Kenya, South Africa, and Zambia (ClinicalTrials.gov number NCT02550067).^6^ The primary goal of the trial was to compare HIV incidence among women randomized to 1 of 3 contraceptive methods (intramuscular depot medroxyprogesterone acetate, copper intrauterine device, or levonorgestrel implant). Overall HIV incidence was 3.81 per 100 person-years, and there were not statistically significant differences between randomized groups. Enrollment began in December 2015 and concluded in September 2017; follow-up concluded in October 2018. Ethics review committees at each study site approved the study protocol, and all participants provided written informed consent.

Women were eligible for the trial if they were HIV seronegative, sexually active, and desiring contraception. At the enrollment visit, plasma was archived to allow retrospective, quantitative HIV RNA PCR testing to assess baseline infection status for those who experienced subsequent HIV seroconversion. After enrollment, participants attended scheduled study follow-up visits every 3 months for a period of 12–18 months, including HIV testing with dual parallel rapid tests. HIV pretest and post-test counseling was conducted according to national guidelines. Participants received a comprehensive package of HIV preventive services at every study visit including HIV risk reduction counselling, syndromic sexually transmitted infection assessment and treatment, and provision of condoms. Toward the end of the trial period, PrEP was offered to participants either by referral or on-site provision by trial staff, in accordance with national guidelines, as PrEP became the standard of care.^7^ Participants who acquired HIV infection were referred for ART and supported in linkage to care.

### Laboratory Methods

HIV serologic testing consisted of 2 rapid HIV tests, run in parallel and using 2 different brands of test kits, conducted at each study site. If one or both of the rapid test results was positive, additional confirmatory testing was conducted with HIV enzyme immunoassay (EIA) (using Abbott ARCHITECT, Abbott Murex or Roche Elecsys HIV combi PT) and HIV RNA polymerase chain reaction (RNA PCR) (Abbott RealTime, Roche COBAS TaqMan, or COBAS AmpliPrep). HIV infection was considered confirmed if HIV EIA was positive and HIV RNA PCR was >400 copies/mL, and participants were considered uninfected if HIV EIA was negative and HIV RNA PCR was not detected. If confirmatory testing was unable to establish or refute infection conclusively, then additional supplemental testing, including retesting of HIV RNA PCR, HIV western blot (BIO-RAD GS HIV-1 Western Blot or Consort E455 with UV transilluminator), and HIV DNA (DNA PCR) (Roche COBAS TaqMan or COBAS AmpliPrep), and if indicated retesting of HIV EIA and/or Geenius (BIO-RAD Geenius HIV 1/2 Supplemental Assay) was conducted, as determined by the trial medical monitor, and final HIV status was determined by a study end points committee. Laboratories followed Good Clinical Laboratory Practice, including regular internal quality control and external quality assurance for the HIV rapid test kits and other HIV tests used throughout the trial.

### Statistical Analysis

We described the number and percentage of visits with true and false positive HIV test results, including among visits with discordant (1 positive and 1 negative) test results or 2 positive test results. Among women determined to have acquired HIV, we described the HIV RNA PCR quantity (copies/mL) using results from the first visit at which HIV infection was detected.

Overall, 11 different HIV rapid test kits were used for testing across the sites. For each test kit, we described the total number of tests performed and the number of false positive and true positive test results using site HIV status determination as the gold standard. We also estimated the false positive rates (FPRs) and the positive predictive values (PPVs) for each test kit. To estimate the 95% confidence intervals for the FPR and PPV, we used the bootstrap method to account for correlation of tests within an individual. Specifically, for each test kit, we first calculated the sampling probability as the proportion of times the test kit was used among all women. We then conducted 1000 simulations, sampling with replacement from the number of times each test kit was used, using the sampling probabilities. For each simulation, we calculated the FPR and PPV, and among the 1000 estimates for each metric, we used the 50th quantile as the point estimate, and the 2.5th and 97.5th quantiles as the lower and upper confidence bounds, respectively. For some of the test kits, the confidence limits for FPR or PPV were inestimable because of zero or very low number of false positive or true positive test results.

We also described advanced testing among women with false positive rapid test results. All analyses were conducted using SAS software (version 9.4; SAS Institute  Inc., Cary, NC)^8^ and R (Version 4.0.2; R Foundation for Statistical Computing, Vienna, Austria).^9^

## RESULTS

A total of 7830 women were enrolled in the study: 5769 (74%) in South Africa, 901 (12%) in Kenya, 658 (8%) in Zambia, and 502 (6%) in Eswatini. Most women (4948, 63%) were younger than 25 years of age.

Among the 7830 women enrolled, 7730 contributed prospective follow-up visits with HIV testing. A total of 48,234 visits (96,468 tests), comprising 10,409 person-years of prospective follow-up, were accrued. By study completion, 412 women became HIV seropositive: 397 acquired HIV during study participation and 15 were retrospectively found to have early, seronegative HIV infection at the time of enrollment; 7318 remained HIV negative. Of the 48,234 visits, dual negative rapid test results occurred at 47,726 (98.9%) visits. Discordant (single positive tests) occurred at 99 (0.2%) visits and dual positive test results at 409 (0.9%) visits. Among the 508 visits with 1 or 2 positive rapid test results, true positive results (ie, confirmed by additional testing) occurred in 412 (81.1%) visits and false positive results occurred in 96 (18.9%) visits (Table 1). The PPV of a positive rapid test result (either single or dual positive) was 81.1%, whereas the PPV was 10.1% for a single positive (discordant) result. The PPV of HIV infection with a positive result defined as dual positive rapid testing was 98.3%.

**TABLE 1.** True and False Positive Rapid Test Results

Description	Number of Visits	Percent
Among visits with 1 or 2 positive rapid test result (N = 508)		
Positive confirmatory results (true positives)	412	81.1
Negative confirmatory results (false positives)	96	18.9
Among visits with 1 positive and 1 negative rapid test result (N = 99)		
Positive confirmatory results (true positives)	10	10.1
Negative confirmatory results (false positives)	89	89.9
Among visits with 2 positive rapid test results (N = 409)		
Positive confirmatory results (true positives)	402	98.3
Negative confirmatory results (false positives)	7	1.7

### True Positive Results

Among the 412 women who were found to have acquired HIV, 402 (97.6%) had dual positive rapid test results, whereas 10 had discordant (1 positive and 1 negative) results. Nearly all (410) had positive EIA results at the time HIV infection was identified, with 2 results being indeterminate. Although high HIV RNA levels often occur during recent infections, we found that HIV RNA levels ranged from undetectable to >1,000,000 copies/mL (Table 2). Twenty-five participants (6.1%) had HIV RNA levels of >1,000,000 copies/mL at the time of detection of infection (with 4 of these having > 10,000,000 copies/mL). Most (356, 86.4%) of initial HIV RNA levels were between 400 and 1,000,000 copies/mL, and 18 (4.4%) had RNA levels that were detectable but ≤400 copies/mL. Notably, 13 (3.2%) had undetected HIV RNA levels at the visit at which HIV seroconversion was detected: 5 reported ART use before the visit (indicating diagnosis outside of the study), 2 did not report ART use but had been lost to follow-up with >1 year since the last negative testing at the study site (thus seroconversion may have been detected after primary infection or ART use misreported), and 6 had no history of ART use or missed visits (with infection diagnosed ∼3 months since last negative HIV testing). For 1 individual, the lower limit of detection was 200 copies/mL for the first assay performed and subsequent testing with a lower level of quantification measured a viral load of 152 copies/mL.

**TABLE 2.** HIV RNA PCR Results Among HIV Seroconverters

Test	Number (%)
HIV RNA PCR (copies/mL) (N = 412)	
Not detected	13 (3.2)
>limit of detection—400	18 (4.4)
401–10,000	110 (26.7)
10,001–100,000	140 (34.0)
100,001–1,000,000	106 (25.7)
>1,000,000	25 (6.1)

Western blot testing was conducted for 36 individuals: 34 were positive and 2 were negative. Of the 2 women with negative results, 1 went on to have a positive result 20 days later and the other was lost to follow-up (EIA was indeterminate and HIV RNA was 256,451 copies/mL). HIV DNA PCR was conducted for 28 individuals: 25 had positive results and 3 were negative (1 had a positive result on follow-up testing 24 days later, 1 had a second negative DNA PCR result 8 days later, and 1 with persistently negative DNA PCR later was suspected to have previously undisclosed ART use). Two individuals had Geenius testing, both positive for HIV-1 and negative for HIV-2.

### False Positive Rapid Test Results

Sixty-two women had false positive rapid HIV test results, occurring at 96 visits; the cumulative probability of a first false positive rapid test at 12 and 18 months were 0.06% and 0.08%, respectively. The proportion of false positive results was higher in visits with discordant (ie, 1 positive and 1 negative) rapid test results, with false positives occurring in 89 of 99 (89.9%) visits with single positive rapid tests, compared with 7 of 409 (1.7%) visits with false positive results when both rapid tests were positive. Fifty-one of the 62 women went on to have at least 1 subsequent visit with HIV testing, and 15 (29.4%) had at least 1 additional occurrence of a false positive result, with 11 of these being at the next consecutive visit.

Three kit types had zero false positive and/or true positive results, and we were not able to estimate their FPRs or PPVs and respective confidence intervals. Of the remaining 8, the FPRs ranged from 0.02% to 0.3%, with PPVs ranging from 75% to 98% (Table 3).

**TABLE 3.** False Positive Rapid Test Results by Rapid Test Kit Type

Kit Type	Number of False Positive Results	Number of True Positive Results	Total Number of Tests	Percent False Positive (95% CI)	Positive Predictive Value (95% CI)
Alere Determine^TM^ HIV-1/2	54	269	35,055	0.15 (0.08, 0.23)	83.3 (76.1, 90.6)
Advanced Quality^TM^ Rapid HIV Test	4	198	18,622	0.02 (0.00, 0.06)	98.0 (94.2, 100)
Uni-Gold^TM^ Recombigen^®^ HIV-1/2	17	131	17,147	0.10 (0.03, 0.20)	88.5 (78.1, 96.7)
Abon™ HIV 1/2/0	12	78	7261	0.17 (0.05, 0.49)	86.7 (65.5, 100)
Premier First Response HIV 1-2.O	7	57	6942	0.10 (0.05, 0.26)	89.1 (71.4, 100.0)
SD Bioline	2	6	4060	0.05 (0.00, 0.35)	75.0 (−, −)†
OraQuick Advance® HIV-1/2*	0	31	3229	0.00 (-, -)†	100.0 (−, −)†
Advanced Quality One Step	2	27	2492	0.08 (0.00, 0.87)	93.1 (33.0, 100.0)
BIOTRACER	5	17	1652	0.30 (0.00, 2.15)	77.3 (0.0, 100.0)
Colloidal Gold HIV(1+2) Antibody*	0	0	7	0.00 (−, −)†	Unable to calculate†
INSTI™ HIV-1 Antibody*	0	0	1	0.00 (−, −)%†	Unable to calculate†
All test kits	103	814	96,468	0.11 (0.07, 0.14)	88.8 (85.4, 91.9)

* FPR and positive predictive values could not be estimated because of zero false positive or zero true positive test results.

† Confidence limits for FPR or PPV were inestimable because of zero or very low number of false positive or true positive test results.

Of the 62 women with false positive rapid test results, 58 had negative EIA results, 2 had indeterminate EIA results, and 2 had positive EIA testing. All 4 women (6.5%) with positive or indeterminate EIA results had single positive rapid test results (Table 4). One was pregnant and used ART for 2 months until confirmatory testing was complete, which included repeat EIA with a different assay (fourth generation), which was negative. Six women (9.7%) had dual false positive rapid test results. One initiated and later stopped ART at an outside facility.

**TABLE 4.** Cases With Either Dual False Positive Rapid Tests or a Single False Positive Rapid Test and Positive/Indeterminate EIA Results

HIV Rapid Test Results	Initial Confirmatory Testing (HIV EIA and HIV RNA)	Additional Confirmatory Testing (Repeat HIV RNA, HIV DNA, HIV Western Blot, Repeat HIV EIA as Needed)	Subsequent HIV Rapid Testing	Other Information
Determine positive First response positive	HIV EIA negative HIV RNA not detected	Repeat HIV RNA not detected HIV DNA negative HIV western blot indeterminate with p31 (1+)	Dual negative HIV rapid tests at 3 subsequent visits including at final visit	Breastfeeding >1-year-old baby, advised to stop (but did not)
Determine positive Unigold positive	HIV EIA negative HIV RNA not detected	Repeat HIV RNA not detected HIV DNA negative HIV western blot negative	Determine positive, First Response negative at 3 subsequent quarterly visits, dual negative HIV rapid tests at final visit	Breastfeeding > 1-year-old baby, advised to stop (but did not)
Determine positive First response positive	HIV EIA negative HIV RNA not detected	Repeat HIV RNA not detected HIV DNA negative HIV western blot indeterminate with ± p55	Dual negative HIV rapid testing at subsequent 3 visits including final visit	
Determine positive Unigold positive	HIV EIA negative HIV RNA not detected	Repeat HIV RNA not detected HIV DNA negative HIV western blot negative	Missed subsequent 2 visits, returned for final visit at which dual HIV rapid tests negative	
Determine positive Advanced quality positive	HIV EIA negative HIV RNA not detected	Repeat HIV RNA not detected HIV DNA negative Western blot negative	Missed next subsequent visit, then dual negative HIV rapid tests at 3 visits including final visit	
Biotracer positive Advanced quality positive	HIV EIA negative HIV RNA not detected	Repeat HIV RNA not detected HIV DNA negative HIV western blot negative	Dual positive HIV rapid tests at subsequent (final) visit, all confirmatory testing was repeated and negative	Initiated ART after dual positive HIV rapid testing at an outside facility. Results provided to care facility and stopped ART. Reported negative subsequent HIV rapid testing
Determine positive First response negative	HIV EIA indeterminate HIV RNA not detected	Repeat HIV RNA not detected HIV DNA negative HIV western blot negative	Three subsequent visits with dual negative HIV rapid testing including final visit	
ABON positive Advanced quality negative	HIV EIA indeterminate HIV RNA not detected	Repeat HIV rapid tests discordant (ABON positive, advanced quality negative) Repeat HIV EIA negative Repeat HIV RNA not detected HIV DNA negative HIV western blot negative	Four additional visits with single false positive HIV rapids, all with negative HIV EIA, including final visit. One intervening visit with dual negative HIV rapid testing	Reported negative testing at a community clinic
Determine negative Unigold positive	HIV EIA positive HIV RNA not detected	Repeat HIV EIA positive, second HIV EIA using different kit negative Repeat HIV RNA not detected HIV DNA negative Western blot indeterminate (1+gp41, 1+p18)	HIV rapid tests negative at next visit and at 2 subsequent visits including final visit	Pregnant, took ART for 2 mo until confirmatory testing and HIV status concluded
Determine positive Unigold negative	HIV EIA positive HIV RNA not detected	Repeat HIV RNA not detected HIV DNA negative HIV western blot indeterminate (±gp160, 1+gp41, ±p31)	Single false positive rapid HIV test at next visit with negative HIV EIA, followed by 3 visits with dual negative HIV rapid testing. Final visit with single false positive rapid testing, HIV EIA indeterminate, HIV Western blot indeterminate, HIV DNA negative, and HIV RNA not detected	

All 62 women with false positive rapid results had undetected RNA levels. Eleven of the 62 had western blot testing, with 7 being negative and 4 indeterminate. Twelve had HIV DNA PCR testing, all negative. In some cases, several rounds of repeated rapid, EIA, and/or additional confirmatory testing were needed to determine the HIV status.

## DISCUSSION

In this large prospective study, involving nearly 50,000 visits with repeated HIV testing of previously HIV seronegative women, we evaluated the frequency and nature of true and false positive rapid HIV testing results. Our findings highlight the challenge that false positive results, which are expected and inherent in any screening test with less than perfect specificity, will occur in programs conducting longitudinal HIV testing.

Rapid testing and associated diagnostic algorithms have made HIV testing accessible globally, resulting in substantial individual and population-level benefits of persons living with HIV knowing their status and initiating ART. To optimize accurate diagnosis of true HIV infections, WHO recommends serial rapid testing: first with a highly sensitive (≥99%) test, with a positive test triggering a second highly specific (≥99%) test. Testing guidelines further include strategies to safeguard against false HIV diagnoses, including the use of approved tests, quality control, use of a third confirmatory rapid or EIA, and the possibility of retesting before ART initiation. For population-based HIV testing programs in high prevalence settings, the public health balance strongly favors obtaining HIV diagnoses over a rare false positive result.

In lower prevalence populations, the relative fraction of false positive results among total positive results will inherently increase. Similarly, groups of recently HIV seronegative persons tested prospectively, as in this study, are effectively a lower-prevalence group, even when those populations live in countries where HIV prevalence and incidence are high. We found that at approximately 1 in 5 visits at which an individual had a positive rapid test, the result was falsely positive, requiring additional confirmatory testing. Notably, the PPV for 2 positive tests (98.3%) was below the WHO goal of >99%, reflecting that, even in this population with a high incidence of HIV, the point prevalence of true positive results when retesting previously HIV seronegative individuals is unlikely to generate a PPV >99%. To maximize detection of incident HIV infections, the trial algorithm conducted dual rapid testing in parallel, and a single positive rapid test was more likely than dual positive tests to be falsely positive, although there were a small number of cases in which both rapids, or 1 rapid and EIA, were positive, with further testing essential to determine a true negative status. Of those with 2 positive tests, 1 initiated ART outside of the study and 2 considered cessation of breastfeeding a child (both over a year old). All 6 were identified by their negative EIA and additional study-based results, and all eventually reverted to negative rapid testing. Of the 66 women with single positive rapid tests, 56 were found to be HIV negative. For some individuals, false positive rapid test results recurred over multiple quarterly follow-up visits. False positive rapid tests may occur because of the presence of cross-reacting antibodies,^10^ which may explain both false positive rapid and EIA results. National algorithms vary with respect to confirmatory testing assays used, but our results reinforce the importance of timely access to confirmatory testing.

In the 4 countries where the Evidence for Contraceptive Options and HIV Outcomes Study was conducted, and others in the region, 2 rapid HIV tests conducted in sequence (a positive screening rapid HIV test followed by a second positive rapid test) is the usual algorithm to confer a diagnosis of HIV infection.^11–14^ Most true positive results met this pattern, although some had only 1 positive rapid or indeterminate EIA results, and an important fraction had low or even undetected HIV RNA levels. Follow-up was quarterly in this study, so infections were generally detected early. These findings emphasize that the textbook pattern of very high HIV RNA and rapid emergence of robustly positive serologic results after HIV acquisition does not occur in all cases.

Our results have important implications for HIV testing for persons taking HIV PrEP. Oral and injectable PrEP agents, when used as prescribed, substantially reduce HIV incidence,^15–17^ and thus the point prevalence of a truly positive result should be very low among PrEP users. The occurrence of false positive results, however, is inherent to the tests used, and thus, it might be expected that most positive rapid test results in consistent PrEP users will be falsely positive.^18^ PrEP research has generally focused on the possibility of false negative results—because of the risk of generation of antiretroviral resistance if PrEP is initiated or continued in someone acquiring HIV—but a false positive result, especially if ART is wrongly initiated, can have substantial and potentially lifelong consequences for individuals. The lifelong consequences of HIV treatment after a misdiagnosis may involve a range of burdensome aspects such as wait time at treatment centers, transportation costs, pill burden, and potential for stigma. Our results also have implications for assessing true HIV seroconversion among PrEP users, as we observed cases of low HIV RNA, indeterminate EIA, and/or negative western blot even in the absence of PrEP.

Limitations of this study include the fact that our results are not directly comparable to most field settings, where rapid tests are conducted in series, and testing is often self-initiated based on perceived risk or known exposures. Rigorously monitored laboratory procedures and test kit storage conditions may also differ from community-based testing.

## CONCLUSIONS

Accuracy in HIV testing is essential. In this prospective study, results were accurate for most visits. However, for individuals who acquired HIV, not all demonstrated the textbook pattern of high HIV RNA levels and clear positive serology. Moreover, although uncommon, false positive HIV rapid tests, sometimes with 2 different tests, were observed. False positive HIV results are more likely to occur with periodic longitudinal testing such as in research settings and/or with PrEP provision, and HIV prevention programs should be aware of the potential for falsely positive results and atypical patterns of positive results and be prepared to provide additional confirmatory testing, particularly with discordant point of care test results.

## APPENDIX. ECHO Trial Consortium

ECHO Trial Consortium: Management Committee: Jared M. Baeten (University of Washington, Seattle, WA), James Kiarie (WHO, Geneva, Switzerland), Timothy D. Mastro (FHI 360, Durham, NC), Nelly R. Mugo (Kenya Medical Research Institute, Nairobi, Kenya; University of Washington, Seattle, WA), Helen Rees (Wits Reproductive Health and HIV Institute, Johannesburg, South Africa). Study Site Principal Investigators: Manzini, Eswatini: Jessica Justman, Zelda Nhlabatsi (Family Life Association of Eswatini & ICAP at Columbia University, New York, NY). Kisumu, Kenya: Elizabeth A. Bukusi, Maricianah Onono (Kenya Medical Research Institute, Nairobi, Kenya). Brits, South Africa: Cheryl Louw (Madibeng Centre for Research). Cape Town, South Africa: Linda-Gail Bekker, Gonasagrie Nair (University of Cape Town and Desmond Tutu HIV Centre). Durban, South Africa: Mags Beksinska, Jennifer Smit (MatCH Research Unit, Faculty of Health Sciences, University of the Witwatersrand). East London, South Africa: G. Justus Hofmeyr, Mandisa Singata-Madliki (University of Fort Hare and University of the Witwatersrand). Edendale: South Africa: Jennifer Smit (MatCH Research Unit, Faculty of Health Sciences, University of the Witwatersrand). Johannesburg, South Africa: Thesla Palanee-Phillips (Wits Reproductive Health and HIV Institute, Faculty of Health Sciences, University of the Witwatersrand). Klerksdorp, South Africa: Raesibe Agnes Pearl Selepe (The Aurum Institute). Ladysmith, South Africa: Sydney Sibiya (Qhakaza Mbokodo Research Clinic). Soshanguve, South Africa: Khatija Ahmed (Setshaba Research Centre). Lusaka, Zambia: Margaret Phiri Kasaro, Jeffrey Stringer (UNC Global Projects Zambia and University of North Carolina at Chapel Hill, Chapel Hill, NC). Other members of the ECHO Trial Consortium: Deborah Baron (Wits Reproductive Health and HIV Institute, Faculty of Health Sciences, University of the Witwatersrand, Johannesburg, South Africa), Deborah Donnell (University of Washington and Fred Hutchinson Cancer Research Center, Seattle, WA), Peter B. Gichangi (International Centre for Reproductive Health—Kenya & Technical University of Mombasa, Mombasa, Kenya), Kate B. Heller (University of Washington, Seattle, WA), Nomthandazo Mbandazayo (Wits Reproductive Health and HIV Institute, Johannesburg, South Africa), Charles S. Morrison (FHI 360, Durham, NC), Kavita Nanda (FHI 360, Durham, NC), Melanie Pleaner (Wits Reproductive Health and HIV Institute, Faculty of Health Sciences, University of the Witwatersrand, Johannesburg, South Africa), Caitlin W. Scoville (University of Washington, Seattle, WA), Kathleen Shears (FHI 360, Washington, DC), Petrus S. Steyn (WHO, Geneva, Switzerland), Douglas Taylor (FHI 360, Durham, NC), Katherine K. Thomas (University of Washington, Seattle, WA), Julia D. Welch (FHI 360, Durham, NC).
